# Dual Energy X-ray Absorptiometry Scanning for Osteoporosis Detection: Analysis of Patients at a Tertiary Care Hospital

**DOI:** 10.7759/cureus.44546

**Published:** 2023-09-01

**Authors:** Aamer Nisar, Hafiz Muhammad Hamza, Ayaz A Awan, Muiz M Malik, Abdullah Gondal, Mehwish Riaz, Hamza Z Bhatti

**Affiliations:** 1 Orthopedic Surgery, Ali Medical Centre, Islamabad, PAK; 2 Medical School, Foundation University School of Health Sciences, Islamabad, PAK; 3 Community Medicine, Foundation University School of Health Sciences, Islamabad, PAK; 4 Biology, Middle East Technical University, Ankara, TUR

**Keywords:** osteoporosis, ultrasound, fragility fractures, bone mineral density (bmd), dexa scan

## Abstract

Introduction: Dual-energy X-ray absorptiometry (DEXA) scanning is a rapid and accurate noninvasive procedure utilized to measure bone mineral density (BMD) and diagnose osteoporosis. The primary objective of this study was to assess the prevalence of osteoporosis in different regions of the body using DEXA scanning in patients attending a tertiary care private hospital. Additionally, we aimed to raise awareness about approved diagnostic methods for osteoporosis.

Methodology: For this retrospective study, a sample size of 384 participants was determined. The selection of participants was based on convenience sampling, considering their availability and accessibility. Data were collected from adult patients aged 18 years and above who underwent DEXA scanning. The information was compiled using Microsoft Excel, obtained from the patient's treating physicians, and evaluated by two medical graduates. Statistical analyses were performed using IBM SPSS Statistics version 26.0 (IBM Corp., Armonk, NY).

Results: The findings unveiled an overarching osteoporosis prevalence of 38.5%, accompanied by distinctive figures of 38.7%, 8.9%, and 38.4% in the lumbar, hip, and forearm regions, respectively. Furthermore, the occurrence of osteopenia was found in 33% of participants in the lumbar region, 35.1% in the hip region, and 39.7% in the forearm region. Additionally, no significant association was found between gender and overall osteoporosis prevalence, suggesting that the susceptibility to osteoporosis did not significantly differ between genders. Moreover, the study emphasized the variations in bone density across different skeletal regions, with the forearm region displaying the lowest mean T-score and Z-score.

Conclusions: The results of this study on osteoporosis prevalence in the lumbar, hip, and forearm regions indicate varying rates among these skeletal sites. Notably, both male and female patients demonstrated an equal susceptibility to developing osteoporosis. Interestingly, the forearm region emerged as the most common site for osteoporosis in males (34.6%), while the lumbar region was the most common in females (41.6%).

## Introduction

Osteoporosis has been described by the consensus development panel of the National Institute of Health (NIH) as a systemic skeletal condition of weakened bones. This condition increases the susceptibility of individuals to bone fractures. Bone strength depends on both bone density and quality [1]. While osteoporosis affects both sexes, women, especially postmenopausal women, are more susceptible to it [2]. Risk factors for primary and secondary osteoporosis can be categorized as modifiable and non-modifiable. Modifiable risk factors, such as low calcium, vitamin D intake, carbonated liquids, low index of body mass, working from a desk job, and prolonged inactivity, can be modified to reduce the risk. On the other hand, non-modifiable risk factors include family history, menopause, and aging [3]. Pakistan ranks fifth globally in terms of osteoporosis prevalence, with an estimated 9.9 million people affected, including 7.2 million women. These numbers are projected to augment to 11.3 million by 2020 and 46 million by the year 2050 [4-5]. A survey indicated that 83% of Pakistani women have vitamin D deficiency, and 72% lead sedentary lifestyles [5]. Despite significant advancements in osteoporosis diagnosis and treatment, there are low rates of osteoporosis assessment and treatment in individuals with fragility fractures internationally [6-8]. Studies using bone densitometry for osteoporosis are also limited, showing minimal utilization of pharmaceutical and lifestyle interventions even in populations with high rates of osteoporosis (35-100%) diagnosis [8, 9]. Challenges to osteoporosis identification and treatment include treatment costs, time and resource requirements for diagnosis, concerns about medications, and a lack of consensus on responsibilities for care provision [8-9]. Dual-energy X-ray absorptiometry (DEXA) scanning is a quick and accurate noninvasive test used to measure bone mineral density (BMD) and diagnose osteoporosis [10]. According to the World Health Organization (WHO), DEXA is the most reliable method for determining bone density [11]. The most commonly examined locations with DEXA are the hip and lower spine, as fractures in these areas can have severe consequences, with a high mortality (20-30%) rate for hip fractures [12]. Vitamin D deficiency is one of the primary risk factors for reduced bone density [13], and its prevalence is higher in Pakistan compared to the USA. Additionally, there is a limited availability of DEXA machines for patient screening in Pakistan, along with a low level of awareness about the significance of osteoporosis [1].

The aim of this study is to assess the osteoporosis prevalence using DEXA scanning in patients presenting to a tertiary care private hospital. Our supplementary goal is to promote understanding about approved gold standard diagnostic methods for osteoporosis. Individuals with low BMD have been provided with a treatment plan that includes lifestyle modifications such as smoking cessation, exercise, and adequate calcium and vitamin D intake.

## Materials and methods

Patients who visited family medicine and polyclinics were the subject of the retrospective cross-sectional investigation at Ali Medical Complex, Islamabad, Pakistan. Data was gathered from January 2019 to December 2022. The sample size was determined using Open Epi software with a 95% confidence level and an anticipated population percentage of 50% in Pakistan having osteoporosis. Considering an absolute precision requirement of 5% and an estimated population size of 1,000,000, the minimum calculated sample size was 384. Convenience sampling was employed to select participants for the study, based on their availability and accessibility. The patients underwent a DEXA scan using Hologic QDR-4500 (Hologic Inc., Waltham, MA, USA). Data was gathered from all adult patients 18 years and over who underwent DEXA scanning. Patients who had supplementary causes of osteoporosis such as osteometabolic disorder, evidence of substantial renal impairment and the existence of advanced-stage cancer, previous history of hip replacement surgery, and prior use of an antiresorptive were excluded.

Digital data from a total of 384 patients were systematically collected. The data collection process involved using Microsoft Excel (Microsoft Corp) to record information gathered by the patient's respective treating physicians. The collected dataset encompassed a comprehensive range of patient characteristics, including age, gender, educational attainment, marital status, and the history of any previous fractures. These detailed attributes were meticulously recorded to ensure a comprehensive understanding of the patient cohort and its potential implications for the study's objectives. All participants were assessed utilizing DEXA and the outcomes were stated as T-scores and Z-scores of the lumbar, hip, and forearm region. T-scores are mean analyses of patients’ BMD with BMD of 30 years of the same sex and ethnicity. Z-scores are mean analysis of patient’s BMD with an average of their own age, sex, and ethnicity. T-scores and Z-scores of below or equivalent to -2.5 standard deviations (SD) at any skeletal site are referred to as osteoporosis as per WHO guidelines. On the other hand, osteopenia is indicated by a T/Z-score between -1 and -2.5 SD. According to this classification, scores of 1 and higher are regarded as normal. The institutional review board of Ali Medical Complex and Foundation University School of Health Sciences ethics committee gave their approval to the proposed study. The privacy and confidentiality of the patients who engaged in this study were maintained. Statistical analyses were conducted using IBM SPSS Statistics version 26.0 (IBM Corp., Armonk, NY). A significance level of p ≤ 0.05 was considered statistically significant. Descriptive statistics were reported, presenting continuous data as mean ± standard deviation (SD) with a 95% confidence interval (CI), and categorical data as frequency and percentage. Chi-squared tests were used for analyzing categorical variables, while independent t-tests were employed for analyzing continuous variables.

## Results

**Figure 1:**
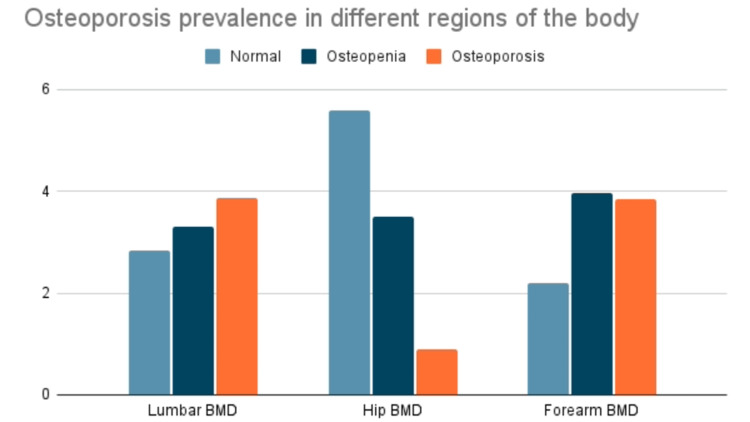
Osteoporosis prevalence in different regions of the body BMD: bone mineral density

We studied the data of 384 individuals in which the mean age of males was 51.72±10.22 years and the mean age of females was 52.49±11.027 years. Within this group 283 were females (73.6%) and 101 were males (26.3%). The findings unveiled an overarching osteoporosis prevalence of 38.5%, accompanied by distinctive figures of 38.7%, 8.9%, and 38.4% in the lumbar, hip, and forearm regions, respectively. Figure 1 demonstrates that the osteoporosis prevalence was 38.7%, 8.9%, and 38.4% while the osteopenia prevalence was 33%, 35.1%, and 39.7% in the lumbar, hip, and forearm regions respectively. 

Table 1 demonstrates the gender-specific patterns of osteoporosis prevalence in all three zones. The most common osteoporotic region for males is the forearm (34.6%) and most common osteoporotic region for females is the lumbar (41.6%).

**Table 1:** Gender-specific patterns of osteoporosis prevalence in three zones

Zones		Osteoporosis	Osteopenia	Normal	P-value
		n (%)	n (%)	n (%)	
Lumbar					
	Male	31 (30.3)	40 (39.2)	31 (30.3)	0.115
	Female	118 (41.6)	87 (30.7)	78 (27.5)	
Hip					
	Male	10( 9.8)	30 (29.4)	62 (60.7)	0.374
	Female	24 (8.57)	104 (37.1)	152 (54.2)	
Forearm					
	Male	35 (34.6)	44 (43.5)	22 (21.7)	0.601
	Female	112 (39.7)	108 (38.2)	62 (21.9)	

No significant association was found between gender and overall osteoporosis prevalence in our study, indicating that the prevalence of osteoporosis did not differ significantly between genders. The findings demonstrate that both male and female patients are equally susceptible to developing osteoporosis in these regions. 

In the lumbar region, the mean T-score was -1.937±1.67, suggesting a lower bone density compared to the reference population. The average Z-scores depicting the lumbar region were -0.973±1.61, indicating a similar trend. Similarly, in the hip region, the mean T-score was -0.909±1.14, while the average Z-scores were -0.209±1.09, both suggesting relatively better bone density compared to the lumbar region. Lastly, in the forearm region, the mean T-score was -2.251±1.44, indicating significantly lower bone density, and the mean Z-score was -1.191±1.32, further supporting this observation. The findings highlight the variations in bone density across different skeletal regions, with the forearm region exhibiting the lowest mean T-score and Z-score.

## Discussion

The goal of our study was to find out the prevalence of osteoporosis in patients presenting to tertiary care hospitals with generalized bone pains and weakness. The osteoporosis prevalence was 38.7%, 8.9%, and 38.4% in the lumbar, hip, and forearm region respectively. There are few epidemiological statistical analyses on osteoporosis in Pakistan because of a shortage of available data and governmental registries. There are few resources accessible for osteoporosis diagnosis, and few DEXA machines that are available in major cities and villages [1]. Existing data from limited hospital-based studies have demonstrated the incidence of osteoporosis through the utilization of sonographic evaluation of the heel but there is a dearth of information regarding BMD utilizing DEXA [14]. The lack of well-established criteria for diagnosing osteoporosis and prescribing treatment based on ultrasound is a significant drawback to employing quantitative ultrasound as a screening technique. Additionally, due to its imprecise nature and the gradual change in bone mass at peripheral sites, ultrasound cannot be relied upon to monitor the progress of women receiving osteoporosis treatment. As a result, the majority of women with a greater risk in sonographic findings will require a DEXA for verification [15] and to demonstrate their requirement for caring in accordance with defined criteria [16] and also to serve as a starting point for therapeutic evaluation [16].

Table 2 shows that there are few studies conducted in Pakistan to address the problem of osteoporosis in women by using the heel sonography method. These studies mainly focus on osteoporosis prevalence in postmenopausal women presented in hospitals of major cities like Karachi, Peshawar, Faisalabad, and Quetta.

**Table 2:** Variations in osteoporosis prevalence in Pakistan; insights from multiple studies

Year	City	Participant	Size	Diagnostic method	Prevalence n (%)	T
2012 [17]	Karachi	Premenopausal/postmenopausal women	1351/65	Heel sonography	Osteopenia (63.8), osteoporotic (17.8) / osteopenia (43.1), osteoporotic (49.3)	-
2011 [18]	Peshawar	Postmenopausal	140	Heel sonography	Osteopenia (43), osteoporotic (27)	−1.6, −2.8
2010 [19]	Peshawar	Postmenopausal from outpatient clinics	240	Heel sonography	Osteopenia (44), osteoporotic (24.5)	-
2010 [20]	Karachi	Premenopausal/postmenopausal visitors (women) to gynecology and obstetrics	170	Heel sonography	Osteopenia (54), osteoporotic (5.6) / osteopenia (47.8), osteoporotic (26.1)	-
2010 [21]	Karachi	Surgery patients with hip fractures	103	Heel sonography	Not known	-
2009 [22]	Faisalabad	Postmenopausal women	300	Heel sonography	Osteopenia (44), osteoporotic (20)	-
2007 [23]	Quetta	Visitors (women) to obstetrics/gynecology unit of Bolan Medical College	334	Heel sonography	Normal (43.7), osteopenia (43.4), osteoporotic (12.9)	−0.2, −1.6, −2.9

Our study's prevalence results differ from those of earlier studies, particularly in terms of osteoporosis. This disparity may be because of varying sample sizes, subject characteristics, study setting, screening technique and the aim of BMD measurements made up of patients. Only women in the postmenopausal phase or participants referred from secondary or tertiary care health facility were enrolled in certain prior study [17-19]. So, there is a need of DEXA scanning in order to know about exact osteoporosis prevalence in Pakistan. Quantitative data is the necessity of the present time to deal with this problem. So, our study is an effort in this aspect.

Osteoporosis is known to have various negative impacts on patients, affecting their physical, mental, and emotional well-being [24]. Fragility fractures associated with osteoporosis are linked to increased rates of morbidity and mortality [25]. Following a fragility fracture, it is possible to experience complications such as fracture-related persistent discomfort and limitations on daily activities [25].

Our study has limitations, particularly given that it is a retrospective study and there may be missing data and unreported variables. In addition, not all confounders were taken into consideration in this investigation. Our study's advantages include its broad subject pool and community-based environment.

## Conclusions

The findings from this study on the osteoporosis prevalence in the lumbar, hip, and forearm regions reveal varying rates among different skeletal sites. The findings unveiled an overarching osteoporosis prevalence of 38.5%. The prevalence was highest in the lumbar region at 38.7%, followed by the forearm region at 38.4%, while the hip region had the lowest prevalence at 8.9%. Importantly, results highlight that both male and female patients have an equal susceptibility to developing osteoporosis. Notably, the most common osteoporotic region for males was the forearm (34.6%), whereas the most common osteoporotic region for females was the lumbar region (41.6%). These findings emphasize the importance of targeted interventions, for example, localized screening and diagnosis, personalized treatment plans, site-specific exercises, and fall prevention strategies. These measures are essential to tackle the burden of osteoporosis in Pakistan, with a specific emphasis on the vulnerable regions identified in this study.
